# Meningeal and Visual Pathway Magnetic Resonance Imaging Analysis after Single and Repetitive Closed-Head Impact Model of Engineered Rotational Acceleration (CHIMERA)-Induced Disruption in Male and Female Mice

**DOI:** 10.1089/neu.2021.0494

**Published:** 2022-06-03

**Authors:** Eileen H. McNamara, Andrew Knutsen, Alexandru Korotcov, Asamoah Bosomtwi, Jiong Liu, Amanda H. Fu, Claire Kostelnik, Antigone A. Grillakis, Haley Spencer, Bernard Dardzinski, Joseph T. McCabe

**Affiliations:** ^1^ Department of Anatomy, Physiology and Genetics, Uniformed Services University of the Health Sciences, Bethesda, Maryland, USA.; ^2^ Center for Neuroscience and Regenerative Medicine, Henry M. Jackson Foundation, Bethesda, Maryland, USA.; ^3^ Department of Anatomy, Physiology and Genetics, Pre-Clinical Behavior and Modeling Core, Uniformed Services University of the Health Sciences, Bethesda, Maryland, USA.

**Keywords:** closed-head impact model of engineered rotational acceleration (CHIMERA), meninges, magnetic resonance imaging (MRI), traumatic brain injury (TBI), traumatic meningeal enhancement (TME), visual pathway

## Abstract

The consequences of forceful rotational acceleration on the central nervous system are not fully understood. While traumatic brain injury (TBI) research primarily has focused on effects related to the brain parenchyma, reports of traumatic meningeal enhancement in TBI patients may possess clinical significance. The objective of this study was to evaluate the meninges and brain for changes in dynamic contrast enhancement (DCE) magnetic resonance imaging (MRI) following closed-head impact model of engineered rotational acceleration (CHIMERA)–induced cerebral insult. Adult male and female mice received one (1 × ; *n* = 19 CHIMERA, *n* = 19 Sham) or four (4 × one/day; *n* = 18 CHIMERA, *n* = 12 Sham) injuries. Each animal underwent three MRI scans: 1 week before injury, immediately after the final injury, and 1 week post-injury. Compared with baseline readings and measures in sham animals, meningeal DCE in males was increased after single impact and repetitive injury. In female mice, DCE was elevated relative to their baseline level after a single impact. One week after CHIMERA, the meningeal enhancement returned to below baseline for single injured male mice, but compared with uninjured mice remained elevated in both sexes in the multiple impact groups. Pre-DCE meningeal T2-weighted relaxation time was increased only after 1 × CHIMERA in injured mice. Since vision is impaired after CHIMERA, visual pathway regions were analyzed through imaging and glial fibrillary acidic protein (GFAP) histology. Initial DCE in the lateral geniculate nucleus (LGN) and superior colliculus (SC) and T2 increases in the optic tract (OPT) and LGN were observed after injury with decreases in DCE and T2 1 week later. Astrogliosis was apparent in the OPT and SC with increased GFAP staining 7 days post-injury. To our knowledge, this is the first study to examine meningeal integrity after CHIMERA in both male and female rodents. DCE-MRI may serve as a useful approach for pre-clinical models of meningeal injury that will enable further evaluation of the underlying mechanisms.

## Introduction

Traumatic brain injury (TBI), including the more common milder forms, are a growing long-term economic and medical burden.^1^ A recent study found there were approximately 27 million new cases of TBI each year and estimated the world prevalence to be around 55.5 million cases.^2^ The most common causes include falls, motor vehicle accidents, and similar impact injuries involving acceleration and deceleration forces.^2,3^ TBI is particularly relevant for military populations and is considered the invisible wound and signature injury from the conflicts in Iraq and Afghanistan.^4^

Repetitive closed-head injuries are also a risk factor for chronic functional impairment and degenerative brain diseases, including chronic traumatic encephalopathy.^5^ There is a potential for sex differences related to higher rates of concussions reported in female collegiate athletes and biomechanical factors, such as reduced head stability.^6,7^ In many species, from invertebrates to mammals, the cranial meninges are a significant structure and act as a protective tissue with stiff physical properties compared with the central nervous system tissue that it encases.^8,9^ While there has been a focus on brain effects, the consequences of impact rotational acceleration on the meninges have received little attention.

Contrast enhancement in magnetic resonance imaging (MRI) by the administration of agents such as gadolinium permit better visualization of the meninges. Human cases of traumatic meningeal enhancement (TME) are only reported in about half of mild TBI patients who receive an MRI.^10,11^ However, TME has been observed, even when other trauma-related imaging changes are not detected.^11–14^ Possible blood–brain barrier damage has also been reported in athletes^15^ and in patients that manifest TME. Little is known about the long-term implications of meningeal changes after injury, but the meninges may be an important site of insult after concussion, a key component in the TBI recovery process, and a therapeutic target for the prevention of subsequent parenchymal damage.^11,16,17^ While the TME mechanism for contrast agent extravasation and accumulation in the meninges is unknown, the clinical ramifications and underlying mechanisms of TME both require investigation.

The focus of this study was to determine the effects of head impact-acceleration upon meningeal integrity as a possible pre-clinical model of TME. In addition, visual impairments have been recognized as a frequent sequela to head impact injuries.^18^ Male and female rodents were included in this study since males are the most common victims of TBI, but more cases of concussion have been reported in female collegiate athletes and biomechanical sex differences have been described in humans.^7,19^ The Closed-Head Impact Model of Engineered Rotational Acceleration (CHIMERA) was selected for this study because of the reliable, non-surgical, and free head rotation design.^20,21^

To identify meningeal changes from contrast agent perfusion after single (1 × ) or repetitive (4 × , separated 24 h apart) CHIMERA, we acquired dynamic contrast enhancement (DCE) magnetic resonance images at Baseline, Day 1 immediately after impact, and 1 week post-injury. Relaxation times of T2-weighted images also were assessed for alterations in inflammation. Since it is well documented that the visual pathway becomes impaired after CHIMERA, the optic tract (OPT), lateral geniculate nucleus (LGN), and superior colliculus (SC) were included for analysis.^22–28^ We hypothesized that murine meninges and brain regions will show increased DCE and T2 immediately after CHIMERA injury and will be sustained after repetitive impacts, indicating meningeal impairment, increased blood–brain barrier permeability, and inflammation. Demonstrating a pre-clinical model of TME is critical for further investigation of this novel marker. Although it has received some attention, clinical studies indicate a significant portion of impact acceleration injuries result in contrast enhancement as a reflection of meningeal impairment. These changes may underlie structural and functional complaints, including edema of the meninges related to headache, and secondary effects from inflammation and disruption of the meningeal barrier.

## Methods

### Animals

A total of 68 7- to 8-week-old male and female C57BL/6J mice (00664) were obtained from the Jackson Laboratory (Bar Harbor, ME) and housed in an Association for Assessment and Accreditation of Laboratory Animal Care International–accredited animal facility for at least 3 days of acclimation before the experiments were started. All procedures were approved by the Uniformed Services University of the Health Sciences Institutional Animal Care and Use Committee. Animals were group-housed (4–5/cage), had access to food and water *ad libitum,* and were maintained on a standard 12 h:12 h light-dark cycle.

### CHIMERA

Animals were randomly assigned to the injured or sham conditions (Table 1). Groups were further divided into either single (1 × , *n* = 19) or repetitive (4 × , 1/day, *n* = 18) CHIMERA-related TBI with appropriate sham counterparts (1 × *n* = 19, 4 × *n* = 12). All mice were anesthetized with isoflurane (3% Forane, Baxter Healthcare Corporation, Deerfield, IL, with 100% O_2_) inside an induction chamber for approximately 2 min. Respiration was monitored, and when deeply unconscious, the mice were transferred to the CHIMERA animal bed and placed in a supine position at a 30° angle with the animal head laid flat horizontally on the head plate. Instrument crosshair markings were used for head alignment for impact of the region surrounding bregma. Velcro straps secured the body but also allowed free rotation of the head and upper torso. During placement, a nose cone on the animal bed delivered isoflurane (2% isoflurane with 100% O_2_) for about 3 min before being removed just prior to impact. For the injured groups, the CHIMERA device piston was driven upward to impact the closed mouse head dorsally (∼0.6 J, 4.9 m/sec velocity). After impact, the mouse was removed from the holder and positioned in a supine orientation in a clean cage to assess the time to return of the righting reflex, a surrogate measurement of return to consciousness. Once the animals turned upright, they were placed back into their home cage. Acetaminophen diluted in water (3% concentration) was administered after injury to all animals. Sham animals were handled identically, including anesthesia treatment of equal duration, placement on the CHIMERA platform, and acetaminophen administration, but were not exposed to the impact.

**Table 1.** MRI Cohort Sample Sizes^*^

Cohort	Injury	Sham	Resolution for T2W (μm^3^)	DCE Resolution (μm^3^)
1	4 × *n* = 12	4 × *n* = 3	150 × 150 × 1000	150 × 150 × 1000
2	4 × *n* = 6	4 × *n* = 9	150 × 150 × 1000	150 × 150 × 1000
3	1 × *n* = 8	1 × *n* = 4	150 × 150 × 1000	150 × 150 × 500
4	1 × *n* = 6	1 × *n* = 8	150 × 150 × 1000	150 × 150 × 500
5	1 × *n* = 5	1 × *n* = 7	150 × 150 × 1000	150 × 150 × 500

^*^ MRI cohort sample sizes.

Pre-contrast and post-contrast T2-weighted images were acquired during each MRI. ProHance gadolinium-based contrast agent was injected during every scan and dynamic contrast enhancement values were acquired with a slightly higher resolution for the single closed-head impact model of engineered rotational acceleration (CHIMERA) experiment versus the repetitive CHIMERA group.

MRI, magnetic resonance imaging.

### MRI acquisition

**FIG. 1.**
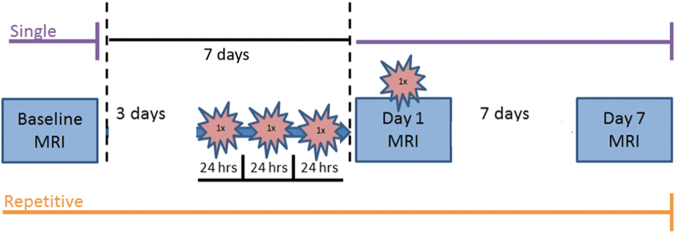
Schematic of the experimental procedure timeline for single (purple, top) and repetitive (orange, bottom) closed-head impact model of engineered rotational acceleration (CHIMERA) experiments. For all groups, three magnetic resonance imaging (MRI) scans were performed at Baseline (1 week before the final CHIMERA), on Day 1 (immediately after the single or last CHIMERA), and Day 7 (1 week following the final injury). The single CHIMERA animals only received one impact immediately before Day 1 scanning, while repetitive CHIMERA mice underwent three impacts separated 24 h apart on the 3 days before the fourth and final impact that was performed immediately before Day 1 scanning. Total scan time was approximately 55 min for each MRI. Regions of interest (ROIs) used for magnetic resonance imaging analyses are displayed in Supplementary Figure S1 and histology ROIs are outlined in Figure 6A. Color image is available online.

*In vivo* images were collected with a Bruker 7T MR scanner (Bruker, Billerica, MA) at three time-points. First, a baseline MRI (Baseline) was conducted 1 week before CHIMERA injuries or sham treatment. The second MRI (Day 1) was initiated within 15 min after the last CHIMERA injury or sham treatment, and the third scan (Day 7) took place 1 week post-injury (Fig. 1). A transmit birdcage coil (Bruker) in combination with an actively decoupled four-channel mouse head receive array coil (Bruker) was used. Pre-contrast and post-contrast T2-weighted images were acquired for each scan. All measurements, T2 and DCE, were acquired during each of the three scans per animal: T2W/PDW/T2 map two-dimensional rapid imaging with refocused echoes repetition time (TR) = 4000 msec; echo time (TE) = 10, 30, 50, 70, 90, 110 msec; echo train length = 2; number of angles (NA) = 2; field of view (FOV) 13.8 × 13.8 mm^2^; matrix 92 × 92; number of slices (NS) = 25; fat suppression; band width (BW) 36 kHz; and time 6:08 min. For DCE three-dimensional fast low angle shot (FLASH), TR/TE = 13/2 msec; flip angle 13°; NA = 1; number of repetitions (NR) = 11; FOV 13.8 × 13.8 × 50 (13.8 × 13.8 × 25 initial cohorts) mm^3^; matrix 92 × 92 × 50(25); no fat suppression; BW 85 kHz; and time 11 min.

Prior to each scan, animals were weighed and placed in an isoflurane anesthesia (4% isoflurane in medical air) induction chamber. A syringe and catheter containing the contrast agent ProHance (Bracco Diagnostics Inc, Milan Italy; 50% Gd in saline) was prepared and the needle tip was directed to the intraperitoneal cavity, but not injected. The mouse was then transferred to the MRI bed, placed in a prone stereotaxic holder for head alignment, and positioned on a circulating water warming pad while maintained under isoflurane through a nose cone. Each MRI session was approximately 55 min in duration and animals were monitored by temperature (maintained 34–37°C) and respiration (maintained 30–70 breaths/min), with appropriate adjustments made to the isoflurane anesthesia (maintained between 1.5–2.0% isoflurane in medical air) while inside the scanner. At the start of the DCE acquisition, ProHance was intraperitoneally injected (0.5 mL 1:1 ProHance:Saline per mouse) with an 11:00 min DCE acquisition time where the injection commenced beginning 2 min into the sequence. Experimenters were blinded to the animal conditions for MRI acquisition and analysis. Following the third scan, animals were euthanized and tissue was collected for histology.

To accommodate time constraints on the treatment sequences, the mice were utilized in cohorts that were divided by single or repetitive injuries and balanced for treatment condition. There was a total of five cohorts. Cohorts 1-2 included 4 × CHIMERA and sham counterparts, while Cohorts 3-5 consisted of 1 × CHIMERA and sham treatment (Table 1). One male 1 × CHIMERA mouse died immediately following impact and the baseline data from this animal was not included in the analysis. One female 4 × CHIMERA mouse displayed hindleg paralysis following the fourth impact. The scan (Day 1) indicated intracerebral hemorrhaging and this mouse was euthanized resulting in no Day 7 scan. Finally, one male 1 × Sham mouse died just before the Day 7 scan, possibly due to anesthesia exposure, and the Day 7 data from this animal was not available.

### Data processing

MRI data were processed using Matlab R2020a (The MathWorks Inc, Natick, MA). T2-relaxation time maps were computed from the multi-echo T2-weighted images. For DCE, the area under the curve (AUC) was calculated to provide a more robust metric for image enhancement.^29^ Details of the image processing are provided in the Supplementary Methods.

MRI data were analyzed for changes in enhancement using a region-of-interest (ROI) approach; which were selected based on previous CHIMERA literature.^21^ ROIs were hand drawn on the pre-contrast 30 msec TE T2-weighted image and on the average of the first three DCE image frames from each scan session using VivoQuant Software version 3.0 (inviCRO LLC, Boston, MA) and ITK-SNAP version 3.8 (Yushkevich, P.A. and Gerig, G., National Institutes of Health [NIH]) software (Supplementary Fig. S1). A bilateral ROI was drawn in the muscle to normalize the data. Animal and voxel exclusion criteria were applied to omit scans with poor contrast uptake and to remove potential errors in ROI drawings (see MRI data processing in the Supplementary Methods). As discussed in the Supplementary Methods, only T2 pre-contrast data was analyzed because T2 pre-contrast is a clinically relevant measurement and is comparable to T2 post-contrast in this study.

### Immunohistochemistry

Immediately following Day 7 MRI scans, mice were anesthetized with a mixture of ketamine and xylazine and then transcardially perfused with cold phosphate buffer solution (0.10 M) and 4% paraformaldehyde in phosphate buffer. Brains were dissected and further fixed in paraformaldehyde for an additional 24 h. They were then transferred to 20% sucrose solution in phosphate buffer for 72 h before freezing the tissue and stored at -80°C until sectioning. A Leica microtome was used to cut 30 μm thick coronal sections. The tissue was stained at least 1 week after sectioning and were washed in Tris-buffered saline with 0.05% triton (TBS-T). Sections were then processed with 0.3% hydrogen peroxide for 30 min and afterwards washed with TBS-T again before blocking buffer (TBS-T with 0.20% triton, 10% goat serum, Vector Laboratories, S-100), and 0.02% bovine serum albumin (BSA; Sigma-Aldrich, A7906) incubation for 1 h at room temperature.

Glial fibrillary acidic protein (GFAP) antibody (1:500; Thermofisher Cat: Ab-6 ASTRO06, Lot: 1376P1810J) was applied to the sections before storage at 4°C overnight. The next day, sections were washed with TBS-T and secondary antibody goat-anti-mouse immunoglobulin G (IgG; 1:500 Jackson Immunoresearch Cat: 115-065-003, Lot: 92050) was applied diluted in blocking buffer for 1 h at room temperature. Sections were again washed with TBS-T before incubation in ABC solution from Vectastain ABC HRP Kit (PK-4000, Vector Laboratories) for 45 min at room temperature. The tissue was washed a final time with TBS-T prior to DAB development with DAB Peroxidase (HRP) Substrate Kit (with nickel), 3,3′-diaminobenzidine (SK-4100, Vector Laboratories) for 3 min. The free-floating sections were mounted onto glass slides and left to dry overnight. Lastly, sections were dehydrated in ethanol gradients (75–100%), cleared in xylene twice, and cover-slipped with Permount mounting media (Fisher Chemical).

#### Visual pathway ROIs

OPT, LGN, and SC were investigated for GFAP staining. Twelve mice (six female and six male mice) per group where successful MRI scans were obtained on all three scans were randomly selected for Carl Zeiss El-Einsatz model #451485 light microscope imaging. Images for the OPT and LGN ROIs were taken at 10 × magnification, whereas the SC were captured at 5 × magnification. To quantify GFAP in each ROI, percent area stained was measured for black/white GFAP images with the threshold function on Image J software (NIH). GFAP values were averaged for three or more sections per animal. Additional regions were investigated, corpus callosum, anterior commissure, and hippocampal fimbria (Supplementary Table S3), but no differences in GFAP were observed.

### Statistical analysis

Graphpad Prism version 8.42 (GraphPad Software, San Diego, CA) was used for figure generation. Data are reported for means or medians, as appropriate, and standard error of the mean (SEM) and coefficient of variation (COV). Statistical analyses were performed using SPSS (IBM, Armonk, NY) and SigmaPlot (Systat Software Inc, San Jose, CA). The body weight data for the 1 × CHIMERA and 1 × Sham groups were analyzed as a two factor (Sex × Day) analysis of variance (ANOVA) and the 4 × CHIMERA and 4 × Sham groups were assessed with a three-factor mixed model ANOVA (Injury × Sex × Day) with Days as a repeated measure. Righting reflex data did not meet the assumptions of normality and was analyzed using separate Kruskal-Wallis ANOVAs for 1 × CHIMERA data and separate analyses each day for the 4 × CHIMERA experiment.

For MRI meningeal analysis, 1 × and 4 × CHIMERA data were analyzed separately with a three-factor linear mixed model analysis (Injury × Sex × Day), with Days treated as a within subjects variable. *Post hoc* pairwise comparisons were made using the Bonferroni correction method. Brain regions were analyzed with a four-factor linear mixed model (Brain Region × Injury × Sex × Day) for the brainstem, cerebellum, cerebral cortex, corpus callosum, dorsal hippocampus, lateral geniculate nucleus, and superior colliculus, and multiple comparisons were evaluated with the Bonferroni correction (Supplementary Methods). The optic tract was analyzed separately for 1 × and 4 × AUC because optic tract mean AUC values greatly differed compared with other brain regions. Since different levels of resolution were performed for 1 × and 4 × cohorts, effect sizes were computed using the Hedge's g formula (equation in Supplementary Methods). The Mann Whitney U-test was used to analyze GFAP histology. GFAP data were also collapsed across sex. A *p* value of ≤0.05 was considered significant.

## Results

CHIMERA velocity and animal body weight were consistent throughout the experiments

The mean maximum energy and the velocity impact for the CHIMERA were consistent across cohort trials and over the days that CHIMERA was performed. The average energy level and piston exit velocity for the cohorts after a single CHIMERA impact (*n* = 19) were 0.60 J (± 0.005 J; COV = 0.15%) and 4.90 m/sec (± 0.02 m/sec; COV = 1.8%). The repetitive CHIMERA experiment (*n* = 18) had average energies and velocities of 0.59 J (± 0.005 J; COV = 0.14%) and 4.87 m/sec (± 0.02 m/sec; COV = 1.7%), respectively, across the days that CHIMERA was performed. Animal body weight across single and repetitive CHIMERA experiments differed between males (*n* = 35) with an average of 24.82 g (± 0.75 g, SEM) and females (*n* = 33) with an average of 19.21 g (± 0.43 g, SEM) as expected. Neither single nor repetitive treatments affected body weights by injury condition; sex differences were the only effect on animal body weight (Supplementary Fig. S2).

### Righting reflex duration increased after CHIMERA and sex differences appeared in the 4 × CHIMERA groups

**FIG. 2.**
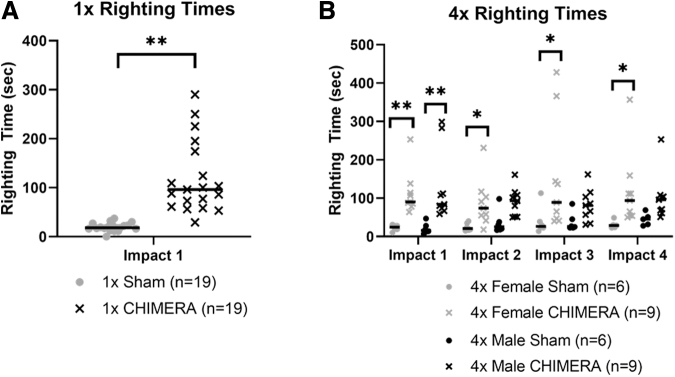
**(A)** Righting times after 1 × closed-head impact model of engineered rotational acceleration (CHIMERA). Injured groups (*n* = 19) for single impact displayed longer righting times and loss of return to consciousness compared with sham counterparts (*n* = 19, H3 = 27.042; *p* < 0.001). **(B)** On Day 1 for 4 × CHIMERA, both male CHIMERA (*n* = 9) and female CHIMERA (*n* = 9) injured mice sustained increased righting reflexes compared with sham (H3 = 23.08; *p* < 0.001). A difference in sex was observed on Days 2-4, where the female 4 × CHIMERA injured animals continued to have longer righting reflex durations compared with female 4 × Sham (*n* = 6). On later days, the male 4 × CHIMERA animals were not different from levels measured in the 4 × Sham male mice (*n* = 6). Bars indicate medians. *p ≤ 0.05; **p ≤ 0.001. In **A**, since no sex differences were identified, the data for male and female were combined in the figure.

Only two brief cases of apnea, about 2 sec, immediately after a single impact were observed in one female and one male. Righting reflex was recorded after animals received anesthesia and sham treatment or anesthesia and CHIMERA (Fig. 2A). Overall, males and females that sustained single and repetitive CHIMERA insults had longer righting reflex times compared with sham animals, suggesting an increase in the return to consciousness from the impacts. The median time for the sham mice to exhibit a righting reflex was 18 sec, while the response in the 1 × CHIMERA-injured animals was significantly longer (H3 = 27.042; *p* < 0.001) with a median time of 96 sec. There were no sex differences so the data were combined in Figure 2A.

Ten instances of apnea divided evenly between males and females were observed across the 72 impact trials in the 4 × CHIMERA group. Apnea mainly occurred following the first impact, but two cases appeared immediately after the third and fourth injuries. Apnea lasted no more than 10 sec in duration. We found that 4 × CHIMERA animals also sustained longer righting times compared with sham mice, on each day they received a CHIMERA injury (Fig. 2B). A Kruskal-Wallis ANOVA was performed on the data for each day, and indicated the median duration of the righting reflex was significantly longer in both male and female 4 × CHIMERA mice on Day 1 (medians: 90 and 84 sec, respectively, H3 = 23.08; *p* < 0.001) than the duration in male and female sham animals (17 and 23 sec, respectively). On later Days 2-4, the female 4 × CHIMERA animals continued to have longer righting reflex durations compared with female sham animals, while the male 4 × CHIMERA animals exhibited longer duration reflex responses, but were not different from levels measured in the 4 × Sham male mice.

### Meninges displayed contrast enhancement at Day 1 following 1 × and 4 × CHIMERA with no apparent changes in the cortex

**FIG. 3.**
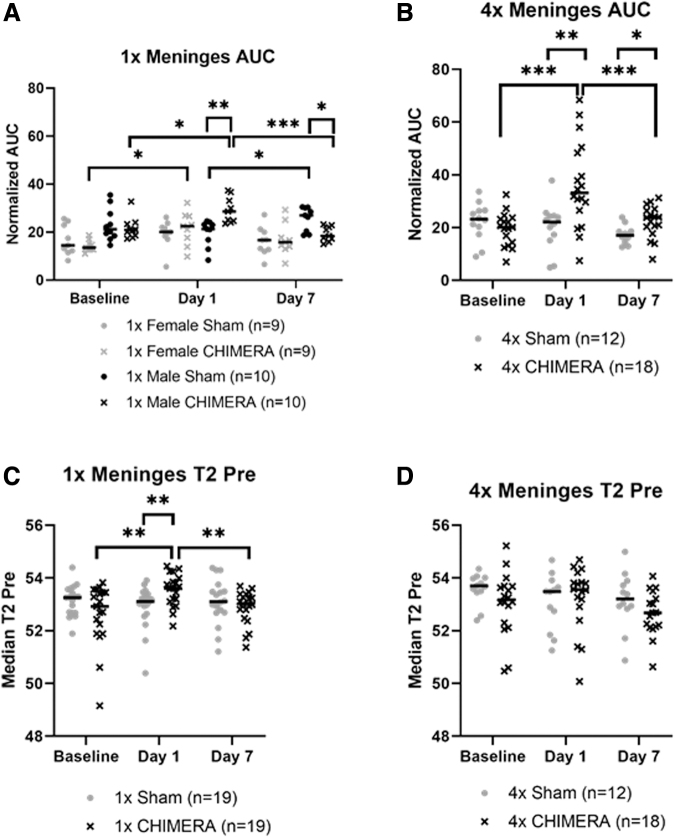
Meningeal magnetic resonance imaging enhancement observed after injury in 1 × closed-head impact model of engineered rotational acceleration (CHIMERA) and 4 × CHIMERA conditions. **(A)** Analysis of the 1 × CHIMERA meningeal regions of interest showed an interaction of Injury × Sex × Day (*p* = 0.006). The male CHIMERA mice (*n* = 10) area under the curve (AUC) measures were significantly greater compared with sham mice (*n* = 10) on Day 1 (*p* = 0.002). CHIMERA animals had elevated AUC values on Day 1 compared with their Baseline for males (*p* = 0.014) and females (*p* = 0.024), on Day 7 injured males demonstrated decreased AUC compared with sham (*p* = 0.022), and within-subject comparison for 1 × CHIMERA males displayed a decrease between Day 1 and Day 7 AUC levels (*p* < 0.001). **(B)** The 4 × CHIMERA experiment revealed an interaction between Injury × Day (*p* = 0.010). On Day 1 after the fourth impact, there was an effect of injury with greater enhancement in the CHIMERA mice (*n* = 18) compared with sham animals (*n* = 12; *p* = 0.005). The CHIMERA group enhancement was also sustained 1 week post-injury (*p* = 0.035). Day 1 CHIMERA were significantly greater than Baseline CHIMERA (*p* < 0.001) and higher than Day 7 CHIMERA (*p* < 0.001). **(C)** T2-relaxation analysis revealed Injury × Day (*p* = 0.009) interaction after 1 × CHIMERA. On Day 1, T2 values increased in the CHIMERA group (*n* = 19) compared with sham mice (*n* = 19; *p* = 0.010). CHIMERA on Day 1 also was greater than CHIMERA at Baseline (*p* = 0.004) or on Day 7 (*p* = 0.007). **(D)** Following 4 × CHIMERA, no significant changes were observed. Bars indicate medians. No sex differences were found after single or repetitive CHIMERA, so male and female results were combined. *p ≤ 0.05; **p ≤ 0.01; ***p ≤ 0.001.

Analysis of AUC changes in the meninges from a single CHIMERA insult indicated there was a significant Injury × Sex × Day interaction effect (F_2, 30.599_ = 6.188; *p* = 0.006). Male 1 × CHIMERA injured mice consistently exhibited greater changes in meninges AUC over time. For the female mice, there was a significant increase in the AUC on Day 1 compared with Baseline (*p* = 0.024) in injured animals, and a similar increase was observed in the male animals (*p* = 0.014). Injured males also demonstrated a greater mean normalized AUC on Day 1 compared with Day 7 (*p* < 0.001). Comparison of between group effects for the males on Day 1 also indicated the 1 × CHIMERA animals exhibited a greater mean AUC than the sham males (*p* = 0.002), but the females at this time were not different due to injury (*p* = 0.453). By Day 7, however, the male 1 × CHIMERA mice showed a significant AUC decrease compared with sham males (*p* = 0.022; Fig. 3A).

Comparison of AUC measures in the meninges after repetitive CHIMERA impacts found a significant two-way Injury × Day interaction (F_2, 25.681_ = 5.613; *p* = 0.010). No sex differences were detected, so the data presents findings collapsed across sex (Fig. 3B). Pairwise comparisons indicated greater enhancement on Day 1 in the mice that sustained CHIMERA injury compared with sham animals (*p* = 0.005). This enhancement difference continued 1 week post-injury, albeit with levels lower than were seen on Day 1, but the difference persisted (*p* < 0.001), and AUC levels for the 4 × CHIMERA mice meninges continued to be greater compared with sham mice on Day 7 (*p* = 0.035). AUC values on Day 1 after CHIMERA injury were also significantly greater than Baseline (*p* < 0.001) and higher than Day 7 (*p* < 0.001). AUC results are summarized in Supplementary Table S1.

**FIG. 4.**
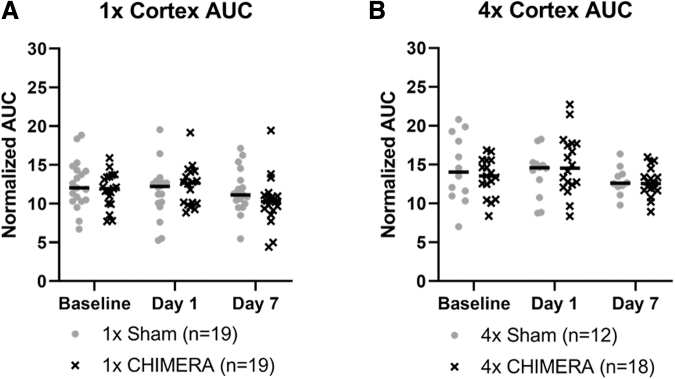
No significant cortical differences in dynamic contrast enhancement, as measured by area under the curve, were observed after **(A)** single or **(B)** repetitive closed-head impact model of engineered rotational acceleration (CHIMERA). No sex differences were observed in the cortex after single or repetitive CHIMERA, so male and female results were combined. AUC, area under the curve.

Since the meninges is a thin structure, there is a chance alterations in DCE are associated with inclusion of underlying cerebral cortex that is captured as a portion of the meninges ROIs. An Injury × Sex × Day analysis indicated the AUC measures of the cerebral cortex directly inferior to the meninges ROIs did not display any significant differences as a function of the injury, sex, or across days. (Fig. 4A). Likewise, no significant changes were observed after 4 × CHIMERA in the cortex over time (Fig. 4B).

Analysis of the 1 × CHIMERA T2-relaxation time data in the meninges (Fig. 3C) found significant two-way interactions of Injury × Day (F_2, 33.699_ = 5.453; *p* = 0.009) and Sex × Day (F_2, 33.699_ = 3.808; *p* = 0.032). However, *post hoc* assessments for differences related to Sex × Day were not different. The Injury × Day difference indicated the average T2 value was greater for the 1 × CHIMERA group than the sham group on Day 1 (*p* = 0.010). The injured and sham groups did not differ at Baseline or on Day 7 (*p* = 0.112). On Day 1, T2 assessment in injured mice were also significantly greater than at Baseline (*p* = 0.004) and Day 7 (*p* = 0.007). The three-way ANOVA of T2 values for the 4 × CHIMERA and sham group indicated no significant meningeal changes (Fig. 3D). T2 results are summarized in Supplementary Table S2.

### Initial enhancement was observed in regions of the visual pathway after CHIMERA followed by decreases 1 week post-injury

**FIG. 5.**
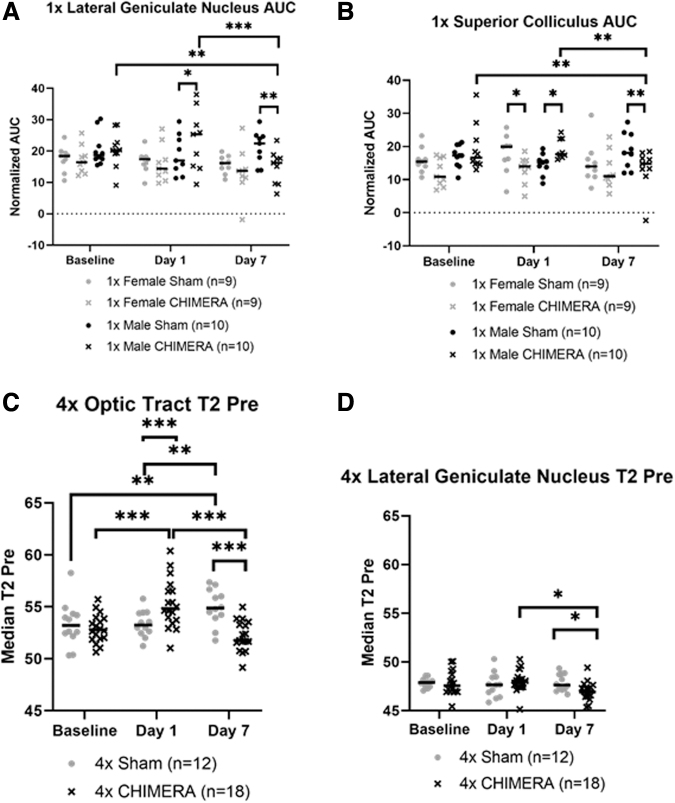
**(A)** The lateral geniculate nucleus (LGN) displayed an effect of injury on Day 1 with an initial increase in area under the curve (AUC) measures after injury in males compared with sham males (*p* = 0.023), but then there was a decrease in AUC on Day 7 (*p* = 0.002). Within-subjects comparison revealed injured males had higher Baseline (*p* = 0.007) and Day 1 (*p* < 0.001) levels compared with Day 7. **(B)** The superior colliculus (SC) showed a similar pattern with an increase in AUC values on Day 1 in 1 × closed-head impact model of engineered rotational acceleration (CHIMERA) males compared with sham (*p* = 0.024) followed by a decrease 1 week post-injury (*p* = 0.008). Injured female mice demonstrated a decrease in AUC measures in the SC on Day 1 compared with sham females (*p* = 0.021). Bonferroni analyses also indicated there was a decrease in AUC on Day 7 for the CHIMERA males compared with measures at Baseline (*p* = 0.002) and Day 1 (*p* = 0.003). **(C)** A pattern of decreased T2 values was observed for injured animals compared with sham on Day 7 for the optic tract (OPT; *p* < 0.001) and LGN (*p* = 0.036). The OPT showed an acute increase in T2 relaxation time on Day 1 for the 4 × CHIMERA mice compared with sham mice (*p* < 0.001). The 4 × CHIMERA animals had higher Day 1 within-group levels compared with Baseline (*p* < 0.001) and Day 7 (*p* < 0.001), whereas the Sham had higher Day 7 within-subject levels compared with Baseline (*p* = 0.001) and Day 1 (*p* = 0.001). **(D)** *Post hoc* analyses for the LGN displayed higher Day 1 compared with Day 7 values for injured animals (*p* = 0.012). Bars indicate medians. *p ≤ 0.05; **p ≤ 0.01; ***p ≤ 0.001. For **(C)** and **(D)**, no sex differences were identified. The data for male and female were combined.

No AUC changes were detected in the OPT. The four-factor ANOVA for 1 × CHIMERA AUC levels in the brain ROIs demonstrated a Brain Region × Injury × Sex × Day interaction (F_18,822.967_ = 1.841; *p* = 0.018). The LGN displayed an effect of injury on Day 1 with an initial AUC increase after injury in males compared with sham mice (*p* = 0.023), but then levels of AUC were decreased on Day 7 (*p* = 0.002). Within-subjects comparisons revealed injured males (Fig. 5A) also had reduced AUC values on Day 7 than measures at Baseline (*p* = 0.007) and Day 1 (*p* < 0.001). The SC showed a similar pattern. Compared with sham animals, AUC in 1 × CHIMERA injured males was increased on Day 1 (*p* = 0.024) followed by a decrease 1 week post-injury (*p* = 0.008). Bonferroni analyses also indicated there was an AUC decrease on Day 7 for CHIMERA males compared with Baseline (*p* = 0.002) and Day 1 (*p* = 0.003; Fig. 5B). Interestingly, injured female mice demonstrated a decrease in mean AUC values compared with sham animals on Day 1 (*p* = 0.021). No significant differences in AUC measures were observed after 4 × CHIMERA injuries.

No significant changes in T2-relaxation were seen after 1 × CHIMERA injury. A four-factor ANOVA of the T2 relaxation measures for the 4 × CHIMERA data indicated there was a significant Brain Region × Injury × Day interaction (F_14,592.324_ = 3.208; *p* < 0.001) and Injury × Sex × Day interaction (F_2,594.253_ = 3.432; *p* = 0.033). The latter interaction effect suggested there was an overall decrease in injured male and female mice across all brain regions on Day 7 compared with sham animals. On Day 1, the OPT exhibited an initial increase in T2-relaxation in the 4 × CHIMERA mice compared with sham animals (*p* < 0.001), and this level in injured mice was greater on Day 1 compared with Baseline (*p* < 0.001) and Day 7 (*p* < 0.001). A pattern of decrease in T2 relaxation time was observed for injured animals compared with sham on Day 7 for the OPT (*p* < 0.001) and LGN (*p* = 0.036)., whereas the sham mice had higher Day 7 within-subject levels compared with Baseline (*p* = 0.001) and Day 1 (*p* = 0.001; Fig. 5C). *Post hoc* analyses for the LGN displayed higher Day 1 compared with Day 7 values for injured animals (*p* = 0.012; Fig. 5D). No T2-relaxation changes were detected in the SC.

### GFAP increases in the visual pathway

**FIG. 6.**
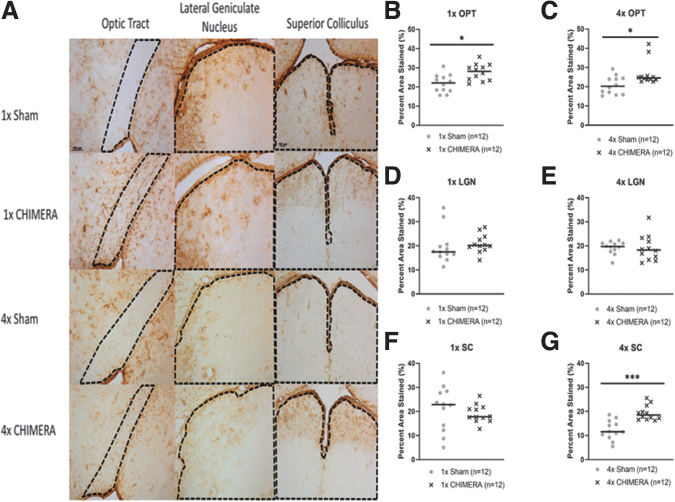
Glial fibrillary acidic protein (GFAP) analyses of the visual pathway. **(A)** Representative GFAP images from the optic tract (OPT), lateral geniculate nucleus (LGN), and superior colliculus (SC; regions are circled with a dashed line). **(B)** Percent area stained analysis for GFAP in the OPT displayed a significant increase in astrogliosis after 1 × closed-head impact model of engineered rotational acceleration (CHIMERA). Following 1 × CHIMERA, there was an increase in GFAP in injured animals (*n* = 12; median value 28.10; U = 28; *p* = 0.0100) compared with sham animals (*n* = 12; median value 22.04). **(C)** After 4 × CHIMERA OPT astrogliosis was also observed in injured mice (*n* = 12; median value 24.55; U = 34; *p* = 0.0284) compared with 4 × Sham mice (median value 20.27). **(D, E)** No significant changes in the LGN after 1 × CHIMERA (sham median = 17.50, 1 × CHIMERA median = 20.15) or 4 × CHIMERA (sham median = 19.71; 4 × CHIMERA median = 18.29). **(F)** GFAP in the SC after 1 × CHIMERA (median = 17.83) was not significantly different than sham (median = 22.87). **(G)** GFAP analysis in the SC showed a significant difference (U = 11; *p* = 0.0001) between 4 × CHIMERA (*n* = 12; median value = 18.53) and sham (*n* = 12; median value = 11.56) with elevated GFAP staining in injured animals 1 week after the fourth impact. *p ≤ 0.05; **p ≤ 0.01; ***p ≤ 0.001. Since no sex differences were identified, the data for male and female were combined in the figures. Color image is available online.

Representative images of GFAP staining are shown in Figure 6A. The OPT exhibited significant changes in astrogliosis following injury. Percent area stained analysis for GFAP in the OPT displayed a significant increase in astrogliosis of injured animals after 1 × CHIMERA (*n* = 12; median value 28.10; U = 28; *p* = 0.0100) compared with sham (median value 22.04; Fig. 6B). Likewise, mice subjected to repetitive impacts had increased GFAP in the OPT (*n* = 12; median value 24.55; U = 34; *p* = 0.0284) compared with 4 × sham mice (median value 20.27; Fig. 6C). GFAP ROIs for the LGN and SC did not display significant differences between 1 × CHIMERA and sham conditions (Fig. 6D, 6F). However, repetitive injury was associated with changes in the SC. Animals that received 4 × CHIMERA impacts (median value = 18.53), compared with sham mice (median value = 11.56) had increased GFAP staining in the SC region (U = 11; *p* = 0.0001; Fig. 6G). Similar to findings after a single injury, no significant changes were observed in the LGN after 4 × CHIMERA (Fig. 6E).

With potential relevance to the meninges, the glial limitans were visually examined at 40 × for qualitative astrocytic changes, but no observable differences between groups were found (data not shown).

## Discussion

### 1 × CHIMERA and 4 × CHIMERA induced a loss of consciousness and sex differences occurred during repetitive impacts

The rationale for the impact sequence was selected to match previous experiments.^21^ The repetitive 4 × CHIMERA design selected chiefly corresponds with other studies in terms of using repeated impacts 24 h apart,^21^ repetitive injuries with similar energy parameters known to result in no or few fatalities,^30,31^ and an energy level (0.6 J) below the threshold of skull fracture, but that still induced changes.^20^

CHIMERA-injured animals exhibited greater durations of loss of consciousness after a single impact compared with the sham mice, but no sex differences were observed. The 4 × CHIMERA group also experienced longer periods of unconsciousness following each impact they received, compared with uninjured matched animals, and a sex difference was observed. Possible reasons for the observed sex difference may be related to CHIMERA biomechanics and body weight. Males were heavier than females, which may pose different injury mechanics or decreased rotational acceleration movement after impact. In a recent repetitive CHIMERA study that used a higher impact level (0.7 J), longer righting reflexes were seen for both injured males and females compared with sham counterparts.^23^ This study may also have relevance for observed body effects with repeated injuries. We did not observe a reduction in body weight in males, in contrast to this previous repetitive TBI study that used the CHIMERA device at 0.7 J, and found a change in males.^23^

### Significant meningeal enhancement occurred after 1 × CHIMERA and 4 × CHIMERA

Meningeal enhancement after injury was observed after both the 1 × and 4 × CHIMERA injury. However, while single and repetitive CHIMERA-exposed groups demonstrated increased AUC values for the meningeal ROI compared with sham counterparts on Day 1, the single CHIMERA mice appeared to return to below AUC Baseline levels 1 week post-injury. This suggests that barrier impairment was resolved a week following single impact, whereas the repetitive CHIMERA mice continued to exhibit elevated enhancement on Day 7, indicating a persistent effect of injury for animals that underwent multiple impacts. Since the slice thickness for the DCE imaging was different for the 4 × (1000 microns) compared with the 1 × (500 microns) cohorts, direct comparisons of the relative change after impact(s) cannot be considered. However, changes relative to the respective sham group AUC levels on Day 1 suggested similar large effect sizes for 1 × CHIMERA male mice and their respective sham group (Hedge's *g* = 1.64) and for the 4 × CHIMERA mice compared with their sham counterparts (Hedge's *g* = 1.10). A similar effect of repeated injury has been seen in a compression injury model in the meninges. After a single injury, meningeal reconstitution was demonstrated with observed revascularization following a week after injury. When a second compression injury was introduced within 1 day of the initial injury, however, meningeal revascularization was impaired.^12^

Resolution of TME suggests meningeal repair and decreased inflammation.^12^ Clinical studies indicate the duration of meningeal enhancement in humans can vary,^17,32,33^ but most TME cases resolve 1 week to 1 month post-injury.^12^ Neuroimaging in the current study after a single injury is consistent with meningeal recovery, but longer time-points post-injury should be included in future studies to better understand the time course of the observed meningeal enhancement, the relationship to injury severity, as well as possible profile differences in responses in male and female animals. In clinical populations, sex differences with respect to TME are also presently unknown. However, the current results with persistent AUC enhancement in injured male mice 7 days after 1 × CHIMERA suggest male meninges may be more sensitive to an impact.^34,35^

The potential sex differences observed in the current pre-clinical data differs from most clinical data suggest that female patients have higher concussion risk and poorer outcomes than males. Female student athletes appear to be particularly susceptible to post-traumatic migraines as well as migraines in general, which could be directly related to meningeal damage. There may also be overall sex differences in the type of symptoms reported after TBI.^34,35^ Additional reports, however, argue that diffusion tensor imaging (DTI) showed more damage and longer recovery in male patients, and that possible baseline sex differences in imaging might be present with hypointense susceptibility weighted images in males compared with females.^36,37^ Further investigation of TBI-related sex differences is needed, as clinical neuroimaging reports consist of small sample sizes and results remain unclear in relation to injury severity.

### Elevated T2 relaxation times in the meninges after impact in the 1 × CHIMERA injured animals

T2-weighted images can inform about robust structural changes. Increased signal related to water content may be indicative of edema and inflammation, whereas hypointensity is associated with hemorrhage.^36,38^ The 1 × CHIMERA injured animals displayed higher T2 values compared with sham mice on Day 1, suggesting edema and inflammation in the meninges after impact, which has been reported to rapidly occur in the minutes and hours after cerebral insult.^39^ By Day 7, however, T2 relaxation times were not significantly different from baseline, suggesting meningeal inflammation had subsided within 1 week post-injury. The lack of T2 change in the meninges after 4 × CHIMERA on Day 1 was surprising, but possibly a result of the longer passage of time (3 days) from initial injury or from the neuroprotective effects of repeated isoflurane exposure during the MRI scans (approximately 1 h) on the days preceding injuries on Days 2-4.^40^ The 4 × CHIMERA animals received 4 consecutive days of isoflurane; therefore, the increased repetitive amount of neuroprotective exposure may have had anti-inflammatory outcomes. Human TME is primarily assessed using T2–fluid-attenuated inversion recovery sequences and only one study has reported some concurrent brain parenchymal T2*-weighted imaging differences in mild TBI patients, but no meningeal T2 relaxation time changes, as used in the current study, have been previously discussed.^10^

### No MR cortical changes were observed

Subsequent to the observed meningeal damage, brain ROIs were also analyzed for indicators of injury and astrogliosis (see Supplementary Methods). Unlike previously described open-head models of TBI, the cortex in this closed-head study did not show MRI contrast agent enhancement.^38,41^ Imaging studies with controlled cortical impact in rats at similar time-points showed T2 and DCE enhancement in the cortex. Corresponding cortical GFAP levels were also increased in one study, but only male rats were examined.^41^ Other closed-head designs, such as lateral impact and weight-drop models, displayed greater damage than the CHIMERA and reported blood–brain barrier damage with contrast agent extravasation into the brain parenchyma. These TBI models appear to mimic severe injury and advanced pathology, whereas the CHIMERA may capture subtle changes.^42,43^

The lack of change in the cortex could be due to the nature of CHIMERA-induced injuries, where here, alterations to the brain parenchymal surface were not apparent *in vivo*. The absence of observable cortical damage *in vivo* is consistent with repetitive CHIMERA studies, which also did not show gross pathology in the cortical tissue near the site of impact.^23^ Likewise, no parenchymal gadolinium extravasation was detected in a clinical study of TME.^17^ The parameters used in this experiment may induce a milder degree of injury relative to MRI visible contusions reported in the clinical scenario.

Finally, coronal measures of DCE in the meninges were obtained at 2 mm caudal to bregma. In addition to assessing the response in this region, the cortex ROI in this study played an additional role as a control for the meningeal ROI. Because of partial volume effects, it was not possible to draw the meningeal ROI at the level of bregma, directly underneath the CHIMERA site of impact. The cortex ROI, however, expanded from bregma to 2 mm caudal to bregma, and did not show any changes, suggesting that the differences observed in the meningeal ROI in corresponding posterior bregma sections reflected changes to the meninges. Edema has been observed through T2 imaging after a pre-clinical model of controlled cortical impact TBI in rats.^38,41^ Consistent with previous CHIMERA imaging studies, however, no T2 changes were reported in the cortex.^31,44^ The absence of T2 enhancement in the cortex, as well as an absence of histological damage, implies that the CHIMERA injury was mild in nature and did not cause significant contusion to the brain parenchymal surface.

### Magnetic resonance alterations in the visual pathway

Differences in magnetic resonance imaging measures were observed as a function of single and multiple insults. The observed pattern of 1 × CHIMERA AUC and 4 × CHIMERA T2-relaxation decreases on Day 7 in these brain regions are consistent with reported susceptibility-weighted imaging hypointensities after concussion.^36^ Previous neuroimaging studies following CHIMERA focused on *ex vivo* DTI, with only one *in vivo* DTI study, and these mainly reported decreases in fractional anisotropy and diffusivity increased in white matter tracts post-injury,^28,44-46^ but no reports of T2 changes were noted.^31,44^ The decreased T2-relaxation observed by Day 7 may indicate a robust anti-inflammatory response or potential hemorrhage 1 week after injury.^38^ In the OPT, Day 7 sham displayed greater T2 intensity than Baseline sham where no change over time was expected. Although the observed MRI differences in the reported brain regions reached significance, the changes were subtle and warrant further investigation to better elucidate the underlying mechanisms.

In parallel with observed differences in the visual system, functional impairment is a frequently reported consequence of CHIMERA injury. The immediate Day 1 MRI changes in areas related to the visual pathway are consistent with previous CHIMERA studies that reported functional impairments on visual tasks, such as the visual cliff test and Morris water maze, as well as decreased visual evoked potentials after single and multiple impacts.^22,26,27^ Visual deficits, however, were observed at later time-points than the current study's reported Day 7 recovery.^22,26^

### GFAP changes in the visual pathway

Increased GFAP staining has been reported at both acute and longer-term time-points after CHIMERA. Although other CHIMERA studies described elevated GFAP changes up to 1 week post-injury in several regions, such as the OPT, corpus callosum, hippocampus, cortex, and SC,^23,30,44,47^ the current study only observed GFAP changes in the OPT and SC (Supplementary Table S3). The LGN is a primary termination for OPT axons and an absence of GFAP staining changes was not expected. This may imply that the astrogliosis in the SC is not the results of optic nerve trauma, but a consequence of the anatomical position of this structure. CHIMERA injury to the parenchyma may affect superficial locations such as OPT and SC that are more prone to injury from displacement. A weight drop study reported increased GFAP in the OPT, LGN, and SC 7 days after injury, but also noted that these regions may have different time lines of inflammation presentation.^48^

A *post hoc* assessment was performed to determine if GFAP staining was associated with AUC or T2 MRI results in corresponding regions. Imaging and histology in this study were not well correlated, with Pearson correlations of 0.01-0.51. Strong imaging and pathology cross-correlates were reported, however, by a different study. Post mortem tissue from TBI patients was imaged using *ex vivo* multidimensional MRI and displayed a strong positive correlation in the corpus callosum with higher multidimensional intensity and increased diffuse axonal injury severity indicated by amyloid precursor protein histology.^49^ Although GFAP in the current study could potentially appear as T2 enhancement because of the inflammation associated with astrogliosis, perhaps the imaging constraints described earlier or the degree of inflammation and lack of edema by Day 7 were factors lending to a difference for what was reported in this clinical report.

### Limitations and future directions

Across the study, the CHIMERA device was found to deliver consistent impacts. There may be a degree of imprecision for the impact location across animals and injuries since the positioning of the scalp above the piston relies on investigator judgement. Nevertheless, the 5 mm piston diameter relative to the dimensions of the mouse scalp suggest head placement on the device is repeatable by an experienced operator. The CHIMERA device is an ideal pre-clinical model. It is a non-surgical procedure allowing a brief exposure to anesthesia at the time of injury, which was essential to the avoidance of meningeal enhancement due to lengthy operative procedures. However, the MRI scanning required prolonged isoflurane use. Although no direct link between isoflurane's effects on contrast agent is evident and the effect of repetitive scans is unclear, anesthetics can alter MRI results by changing animal body temperature and metabolism. Many variables can affect imaging and hypothermia due to anesthetics is a primary concern.^50^ A recent study showed that isoflurane exposure during MRI reduced histological blood–brain barrier damage compared with animals that did not undergo prolonged sedation.^51^ The MRI differences observed in some sham animals could be related to these biological variable responses to isoflurane since individual differences in anesthetic stabilization were observed despite standardized imaging procedures/setup. Future studies could include the use of alternative sedation agents as a substitute for isoflurane during imaging.

MRI analysis was restricted by image resolution. No initial whole–brain analyses was conducted because of the slice thickness, so that voxel-based analysis was not optimal. Acquisition resolution resulted in deeper and more medial ROIs being more susceptible to partial volume effects during MRI analysis. A surface coil was utilized throughout the current study; therefore signal-to-noise was decreased further from the coil. Like the brain, the skull and underlying meninges are heterogeneous with variability in thickness depending on the location and species measured. Studies of murine meninges report inter-subject and tissue preparation variability, adding to the complexity in measuring meningeal thickness.^9,52-54^ While composition is fairly consistent across species, dural thickness has been reported to be about 50 μm in rats compared with approximately 564 μm in humans, but variability in tissue preservation methods can greatly alter this measurement.^52,55^

There may also be differences among rodent meningeal structure with well characterized subarachnoid space reported in rats, whereas mice may not possess such space or arachnoid trabeculae.^54,56^ This variability in meningeal thickness and its size relative to the MRI resolution means that the meninges ROI contains some other structures; however, the cortex ROI provided a control to differentiate between the meninges and underlying brain parenchyma. A rodent meningeal model is ideal in terms of logistics, but future TME study should include larger animal models that more closely resemble human meningeal properties.^57^ The current study employed an intraperitoneal route for contrast agent injection. The intraperitoneal route has been employed previously in rodent blast studies,^58^ and has been seen as the better alternative when the study requires multiple injection events.^59,60^ While intravenous administration is more clinically relevant, tail vein access is challenging and repetitive local access is an effective alternative with lessened local vessel damage

While the resolution of MRI was at the limits for exclusive capture of voxels that were circumscribed to the meningeal layer, the results suggest that a pre-clinical model of TME can be achieved using the CHIMERA device, which can allow for further study to determine the mechanism of action. To understand the mechanisms relevant to TME and potential protective meningeal properties, *ex vivo* analysis of the meninges could provide detailed structural data to show anatomical changes in the meninges after injury.^11,17,32,52,61–65^ Additional approaches, such as meningeal two-photon microscopy, has been successfully employed and could assist in studying the pathway for neuroimmune exchange in the meninges that contribute to the observed meningeal enhancement.^8,11,12,66^ Lastly, determining comparable differences between single and repetitive injury and potential behavioral deficits related to meningeal damage is critical to defining functional impairments. TME in humans may provide a sensitive imaging biomarker for mild TBI and could present a therapeutic target to prevent additional inflammatory damage to the parenchyma.

## Supplementary Material

Supplemental data

Supplemental data

Supplemental data

Supplemental data

Supplemental data

Supplemental data

## References

[1] Hyder, A.A., Wunderlich, C.A., Puvanachandra, P., Gururaj, G., and Kobusingye, O.C. (2007). The impact of traumatic brain injuries: a global perspective. NeuroRehabilitation 22, 341–353. 18162698

[2] James, S.L., Theadom, A., Ellenbogen, R.G., Bannick, M.S., Montjoy-Venning, W., Lucchesi, L.R., Abbasi, N., Abdulkader, R., Abraha, H.N., and Adsuar, J.C. (2019). Global, regional, and national burden of traumatic brain injury and spinal cord injury, 1990–2016: a systematic analysis for the Global Burden of Disease Study 2016. Lancet Neurol. 18, 56–87. 30497965 10.1016/S1474-4422(18)30415-0 PMC6291456

[3] Faul, M., Wald, M.M., Xu, L., and Coronado, V.G. (2010). Traumatic Brain Injury in the United States; Emergency Department Visits, Hospitalizations, and Deaths, 2002-2006. Centers for Disease Control and Prevention; National Center for Injury Prevention and Control: Atlanta, GA.

[4] Tanielian, T., Haycox, L.H., Schell, T.L., Marshall, G.N., Burnam, M.A., Eibner, C., Karney, B.R., Meredith, L.S., Ringel, J.S., and Vaiana, M.E. (2008). Invisible Wounds of War. Summary and Recommendations for Addressing Psychological and Cognitive Injuries. Rand Corp: Santa Monica, CA.

[5] McKee, A.C., Alosco, M.L., and Huber, B.R. (2016). Repetitive head impacts and chronic traumatic encephalopathy. Neurosurg. Clin. N. Am. 27, 529–535. 27637402 10.1016/j.nec.2016.05.009 PMC5028120

[6] Covassin, T., Moran, R., and Elbin, R. (2016). Sex differences in reported concussion injury rates and time loss from participation: an update of the National Collegiate Athletic Association Injury Surveillance Program from 2004–2005 through 2008–2009. J. Athl. Train. 51, 189–194. 26950073 10.4085/1062-6050-51.3.05 PMC4852524

[7] Tierney, R.T., Sitler, M.R., Swanik, C.B., Swanik, K.A., Higgins, M., and Torg, J. (2005). Gender differences in head-neck segment dynamic stabilization during head acceleration. Med. Sci. Sports Exerc. 37, 272–279. 15692324 10.1249/01.mss.0000152734.47516.aa

[8] Kierdorf, K., Masuda, T., Jordão, M.J.C., and Prinz, M. (2019). Macrophages at CNS interfaces: ontogeny and function in health and disease. Nat. Rev. Neurosci. 20, 547–562. 31358892 10.1038/s41583-019-0201-x

[9] Maikos, J.T., Elias, R.A., and Shreiber, D.I. (2008). Mechanical properties of dura mater from the rat brain and spinal cord. J. Neurotrauma 25, 38–51. 18355157 10.1089/neu.2007.0348

[10] Chiara Ricciardi, M., Bokkers, R.P., Butman, J.A., Hammoud, D.A., Pham, D.L., Warach, S., and Latour, L.L. (2017). Trauma-specific brain abnormalities in suspected mild traumatic brain injury patients identified in the first 48 h after injury: a blinded magnetic resonance imaging comparative study including suspected acute minor stroke patients. J. Neurotrauma 34, 23–30. 27215444 10.1089/neu.2015.4338 PMC5198056

[11] Roth, T.L., Nayak, D., Atanasijevic, T., Koretsky, A.P., Latour, L.L., and McGavern, D.B. (2014). Transcranial amelioration of inflammation and cell death after brain injury. Nature 505, 223. 24317693 10.1038/nature12808 PMC3930079

[12] Russo, M.V., Latour, L.L., and McGavern, D.B. (2018). Distinct myeloid cell subsets promote meningeal remodeling and vascular repair after mild traumatic brain injury. Nat. Immunol. 19, 442. 29662169 10.1038/s41590-018-0086-2 PMC6426637

[13] Castro, M.A., Williford, J.P., Cota, M.R., MacLaren, J.M., Dardzinski, B.J., Latour, L.L., Pham, D.L., and Butman, J.A. (2016). Quantification of traumatic meningeal injury using dynamic contrast enhanced (DCE) fluid-attenuated inversion recovery (FLAIR) imaging, in: *Medical Imaging 2016: Biomedical Applications in Molecular, Structural, and Functional Imaging*. International Society for Optics and Photonics: Bellingham, WA, p. 97882P.

[14] Davis, T.S., Nathan, J.E., Tinoco Martinez, A.S., De Vis, J.B., Turtzo, L.C., and Latour, L.L. (2020). Comparison of T1-post and FLAIR-post MRI for identification of traumatic meningeal enhancement in traumatic brain injury patients. PloS One 15, e0234881. 32614835 10.1371/journal.pone.0234881 PMC7332069

[15] O'Keeffe, E., Kelly, E., Liu, Y., Giordano, C., Wallace, E., Hynes, M., Tiernan, S., Meagher, A., Greene, C., and Hughes, S. (2020). Dynamic blood-brain barrier regulation in mild traumatic brain injury. J. Neurotrauma 37, 347–356. 31702476 10.1089/neu.2019.6483 PMC10331162

[16] Coles, J.A., Myburgh, E., Brewer, J.M., and McMenamin, P.G. (2017). Where are we? The anatomy of the murine cortical meninges revisited for intravital imaging, immunology, and clearance of waste from the brain. Prog. Neurobiol. 156, 107–148. 10.1016/j.pneurobio.2017.05.002 28552391

[17] Turtzo, L.C., Jikaria, N., Cota, M.R., Williford, J.P., Uche, V., Davis, T., MacLaren, J., Moses, A.D., Parikh, G., and Castro, M.A. (2020). Meningeal blood-brain barrier disruption in acute traumatic brain injury. Brain Commun. 2, fcaa143. 10.1093/braincomms/fcaa143 PMC7869431 33829156

[18] Goodrich, G.L., Martinsen, G.L., Flyg, H.M., Kirby, J., Garvert, D.W., and Tyler, C.W. (2014). Visual function, traumatic brain injury, and posttraumatic stress disorder. J. Rehabil. Res. Dev. 51, 547–558. 25144168 10.1682/JRRD.2013.02.0049

[19] Covassin, T., Elbin, R., Kontos, A., and Larson, E. (2010). Investigating baseline neurocognitive performance between male and female athletes with a history of multiple concussion. J. Neurol. Neurosurg. Psychiatry 81, 597–601. 20522868 10.1136/jnnp.2009.193797

[20] Namjoshi, D.R., Cheng, W.H., McInnes, K.A., Martens, K.M., Carr, M., Wilkinson, A., Fan, J., Robert, J., Hayat, A., and Cripton, P.A. (2014). Merging pathology with biomechanics using CHIMERA (closed-head impact model of engineered rotational acceleration): a novel, surgery-free model of traumatic brain injury. Mol. Neurodegener. 9, 55. 25443413 10.1186/1750-1326-9-55 PMC4269957

[21] McNamara, E.H., Grillakis, A.A., Tucker, L.B., and McCabe, J.T. (2020). The closed-head impact model of engineered rotational acceleration (CHIMERA) as an application for traumatic brain injury pre-clinical research: a status report. Exp. Neurol., 113409. 32692987 10.1016/j.expneurol.2020.113409

[22] Desai, A., Chen, H., and Kim, H.Y. (2020). Multiple mild traumatic brain injuries lead to visual dysfunction in a mouse model. J. Neurotrauma 37, 286–294. 31530220 10.1089/neu.2019.6602 PMC6964804

[23] Tucker, L.B., Fu, A.H., and McCabe, J.T. (2021). Hippocampal-dependent cognitive dysfunction following repeated diffuse rotational brain injury in male and female mice. J. Neurotrauma 38, 1585–1606. 33622092 10.1089/neu.2021.0025 PMC8126427

[24] Cheng, W.H., Martens, K.M., Bashir, A., Cheung, H., Stukas, S., Gibbs, E., Namjoshi, D.R., Button, E.B., Wilkinson, A., Barron, C.J., Cashman, N.R., Cripton, P.A., and Wellington, C.L. (2019). CHIMERA repetitive mild traumatic brain injury induces chronic behavioural and neuropathological phenotypes in wild-type and APP/PS1 mice. Alzheimers Res. Ther. 11, 6. 30636629 10.1186/s13195-018-0461-0 PMC6330571

[25] Vonder Haar, C., Martens, K.M., Bashir, A., McInnes, K.A., Cheng, W.H., Cheung, H., Stukas, S., Barron, C., Ladner, T., Welch, K.A., Cripton, P.A., Winstanley, C.A., and Wellington, C.L. (2019). Repetitive closed-head impact model of engineered rotational acceleration (CHIMERA) injury in rats increases impulsivity, decreases dopaminergic innervation in the olfactory tubercle and generates white matter inflammation, tau phosphorylation and degeneration. Exp. Neurol. 317, 87–99. 30822421 10.1016/j.expneurol.2019.02.012

[26] Chen, H., Kevala, K., Aflaki, E., Marugan, J., and Kim, H.Y. (2021). GPR110 ligands reduce chronic optic tract gliosis and visual deficit following repetitive mild traumatic brain injury in mice. J. Neuroinflammation 18, 157. 34273979 10.1186/s12974-021-02195-y PMC8286622

[27] Desai, A., Chen, H., Kevala, K., and Kim, H.Y. (2021). Higher n-3 polyunsaturated fatty acid diet Improves long-term neuropathological and functional outcome after repeated mild traumatic brain injury. J. Neurotrauma 38, 2622–2632. 33913741 10.1089/neu.2021.0096 PMC8403198

[28] Reyes, L.D., Haight, T., Desai, A., Chen, H., Bosomtwi, A., Korotcov, A., Dardzinski, B., Kim, H.Y., and Pierpaoli, C. (2020). Investigation of the effect of dietary intake of omega-3 polyunsaturated fatty acids on trauma-induced white matter injury with quantitative diffusion MRI in mice. J. Neurosci. Res. 98, 2232–2244. 32840025 10.1002/jnr.24705 PMC7589213

[29] Iliff, J.J., Lee, H., Yu, M., Feng, T., Logan, J., Nedergaard, M., and Benveniste, H. (2013). Brain-wide pathway for waste clearance captured by contrast-enhanced MRI. J. Clin. Invest. 123, 1299–1309. 23434588 10.1172/JCI67677 PMC3582150

[30] Chen, H., Desai, A., and Kim, H.Y. (2017). Repetitive closed-head impact model of engineered rotational acceleration induces long-term cognitive impairments with persistent astrogliosis and microgliosis in mice. J. Neurotrauma 34, 2291–2302. 28288551 10.1089/neu.2016.4870 PMC5510798

[31] Nolan, A., Hennessy, E., Krukowski, K., Guglielmetti, C., Chaumeil, M.M., Sohal, V.S., and Rosi, S. (2018). Repeated mild head injury leads to wide-ranging deficits in higher-order cognitive functions associated with the prefrontal cortex. J. Neurotrauma 35, 2425–2434. 29732949 10.1089/neu.2018.5731 PMC6196749

[32] Kim, S.C., Park, S.-W., Ryoo, I., Jung, S.C., Yun, T.J., Choi, S.H., Kim, J.-h., and Sohn, C.-H. (2014). Contrast-enhanced FLAIR (fluid-attenuated inversion recovery) for evaluating mild traumatic brain injury. PLoS One 9, e102229. 25028975 10.1371/journal.pone.0102229 PMC4100883

[33] Elster, A.D., and DiPersio, D.A. (1990). Cranial postoperative site: assessment with contrast-enhanced MR imaging. Radiology 174, 93–98. 2294578 10.1148/radiology.174.1.2294578

[34] Koerte, I.K., Schultz, V., Sydnor, V.J., Howell, D.R., Guenette, J.P., Dennis, E., Kochsiek, J., Kaufmann, D., Sollmann, N., and Mondello, S. (2020). Sex-related differences in the effects of sports-related concussion: a review. J. Neuroimaging 30, 387–409. 32533752 10.1111/jon.12726 PMC8221087

[35] Mihalik, J.P., Register-Mihalik, J., Kerr, Z.Y., Marshall, S.W., McCrea, M.C., and Guskiewicz, K.M. (2013). Recovery of posttraumatic migraine characteristics in patients after mild traumatic brain injury. Am. J. Sports Med. 41, 1490–1496. 23696213 10.1177/0363546513487982

[36] Helmer, K.G., Pasternak, O., Fredman, E., Preciado, R.I., Koerte, I.K., Sasaki, T., Mayinger, M., Johnson, A.M., Holmes, J.D., and Forwell, L.A. (2014). Hockey Concussion Education Project, Part 1. Susceptibility-weighted imaging study in male and female ice hockey players over a single season. J. Neurosurg. 120, 864–872. 10.3171/2013.12.JNS132093 PMC4450742 24490839

[37] Fakhran, S., Yaeger, K., Collins, M., and Alhilali, L. (2014). Sex differences in white matter abnormalities after mild traumatic brain injury: localization and correlation with outcome. Radiology 272, 815–823. 24802388 10.1148/radiol.14132512

[38] Li, W., Watts, L., Long, J., Zhou, W., Shen, Q., Jiang, Z., Li, Y., and Duong, T.Q. (2016). Spatiotemporal changes in blood-brain barrier permeability, cerebral blood flow, T2 and diffusion following mild traumatic brain injury. Brain Res. 1646, 53–61. 27208495 10.1016/j.brainres.2016.05.036

[39] Bareyre, F., Wahl, F., McIntosh, T.K., and Stutzmann, J.-M. (1997). Time course of cerebral edema after traumatic brain injury in rats: effects of riluzole and mannitol. J. Neurotrauma 14, 839–849. 9421455 10.1089/neu.1997.14.839

[40] Statler, K.D., Alexander, H., Vagni, V., Holubkov, R., Dixon, C.E., Clark, R.S., Jenkins, L., and Kochanek, P.M. (2006). Isoflurane exerts neuroprotective actions at or near the time of severe traumatic brain injury. Brain Res. 1076, 216–224. 16473332 10.1016/j.brainres.2005.12.106

[41] Yu, M., Wang, M., Yang, D., Wei, X., and Li, W. (2019). Dynamics of blood brain barrier permeability and tissue microstructure following controlled cortical impact injury in rat: a dynamic contrast-enhanced magnetic resonance imaging and diffusion kurtosis imaging study. Magn. Reson. Imaging. 62, 1–9. 30660704 10.1016/j.mri.2019.01.017

[42] Tagge, C.A., Fisher, A.M., Minaeva, O.V., Gaudreau-Balderrama, A., Moncaster, J.A., Zhang, X.-L., Wojnarowicz, M.W., Casey, N., Lu, H., and Kokiko-Cochran, O.N. (2018). Concussion, microvascular injury, and early tauopathy in young athletes after impact head injury and an impact concussion mouse model. Brain 141, 422–458. 29360998 10.1093/brain/awx350 PMC5837414

[43] Veksler, R., Vazana, U., Serlin, Y., Prager, O., Ofer, J., Shemen, N., Fisher, A.M., Minaeva, O., Hua, N., and Saar-Ashkenazy, R. (2020). Slow blood-to-brain transport underlies enduring barrier dysfunction in American football players. Brain 143, 1826–1842. 32464655 10.1093/brain/awaa140 PMC7297017

[44] Haber, M., Hutchinson, E., Sadeghi, N., Cheng, W., Namjoshi, D., Cripton, P., Irfanoglu, M., Wellington, C., Diaz-Arrastia, R., and Pierpaoli, C. (2017). Defining an analytic framework to evaluate quantitative MRI markers of traumatic axonal injury: preliminary results in a mouse closed head injury model. eNeuro 4, ENEURO.0164-0117.2017. 10.1523/ENEURO.0164-17.2017 PMC5616192 28966972

[45] Komlosh, M.E., Benjamini, D., Hutchinson, E.B., King, S., Haber, M., Avram, A.V., Holtzclaw, L.A., Desai, A., Pierpaoli, C., and Basser, P.J. (2018). Using double pulsed-field gradient MRI to study tissue microstructure in traumatic brain injury (TBI). Microporous Mesoporous Mater. 269, 156–159. 30337835 10.1016/j.micromeso.2017.05.030 PMC6188654

[46] Sauerbeck, A.D., Fanizzi, C., Kim, J.H., Gangolli, M., Bayly, P.V., Wellington, C.L., Brody, D.L., and Kummer, T.T. (2018). modCHIMERA: a novel murine closed-head model of moderate traumatic brain injury. Sci. Rep. 8, 7677. 29769541 10.1038/s41598-018-25737-6 PMC5955903

[47] Li, H., Zhang, C., Yang, C., Blevins, M., Norris, D., Zhao, R., and Huang, M. (2020). C-terminal binding proteins 1 and 2 in traumatic brain injury-induced inflammation and their inhibition as an approach for anti-inflammatory treatment. Int. J. Biol. Sci. 16, 1107–1120. 32174788 10.7150/ijbs.42109 PMC7053329

[48] Evanson, N.K., Guilhaume-Correa, F., Herman, J.P., and Goodman, M.D. (2018). Optic tract injury after closed head traumatic brain injury in mice: a model of indirect traumatic optic neuropathy. PLoS One 13, e0197346. 29746557 10.1371/journal.pone.0197346 PMC5944994

[49] Benjamini, D., Iacono, D., Komlosh, M.E., Perl, D.P., Brody, D.L., and Basser, P.J. (2021). Diffuse axonal injury has a characteristic multidimensional MRI signature in the human brain. Brain 144, 800–816. 33739417 10.1093/brain/awaa447 PMC8041044

[50] Hildebrandt, I.J., Su, H., and Weber, W.A. (2008). Anesthesia and other considerations for in vivo imaging of small animals. ILAR J. 49, 17–26. 18172330 10.1093/ilar.49.1.17

[51] Dhaya, I., Griton, M., and Konsman, J.P. (2021). Magnetic resonance imaging under isoflurane anesthesia alters cortical cyclooxygenase-2 expression and glial cell morphology during sepsis-associated neurological dysfunction in rats. Animal Model Exp. Med. 4, 249–260. 34557651 10.1002/ame2.12167 PMC8446714

[52] Walsh, D.R., Zhou, Z., Li, X., Kearns, J., Newport, D.T., and Mulvihill, J.J. (2021). Mechanical properties of the cranial meninges: a systematic review. J. Neurotrauma 38, 1748–1761. 33191848 10.1089/neu.2020.7288

[53] Fabris, G., Suar, Z.M., and Kurt, M. (2019). Micromechanical heterogeneity of the rat pia-arachnoid complex. Acta Biomater. 100, 29–37. 31585202 10.1016/j.actbio.2019.09.044

[54] Oda, Y., and Nakanishi, I. (1984). Ultrastructure of the mouse leptomeninx. J. Comp. Neurol. 225, 448–457. 6202729 10.1002/cne.902250310

[55] Kinaci, A., Bergmann, W., Bleys, R.L., van der Zwan, A., and van Doormaal, T.P. (2020). Histologic comparison of the dura mater among species. Comp. Med. 70, 170–175. 32014084 10.30802/AALAS-CM-19-000022 PMC7137549

[56] Saboori, P. (2021). Subarachnoid space trabeculae architecture. Clin. Anat. 34, 40–50. 32519396 10.1002/ca.23635

[57] Cullen, D.K., Harris, J.P., Browne, K.D., Wolf, J.A., Duda, J.E., Meaney, D.F., Margulies, S.S., and Smith, D.H. (2016). A porcine model of traumatic brain injury via head rotational acceleration, in: Injury Models of the Central Nervous System. F. Kobeissy, C. Dixon, R. Hayes, and S. Mondello (eds). Humana Press: New York, NY, pps. 289–324. 10.1007/978-1-4939-3816-2_17 PMC5553045 27604725

[58] Rubovitch, V., Ten-Bosch, M., Zohar, O., Harrison, C.R., Tempel-Brami, C., Stein, E., Hoffer, B.J., Balaban, C.D., Schreiber, S., and Chiu, W. T. (2011). A mouse model of blast-induced mild traumatic brain injury. Exper. Neurol. 232, 280–289. 21946269 10.1016/j.expneurol.2011.09.018 PMC3202080

[59] Portnoy, S., Bishop, J., Dazai, J., Spring, S., and Henkelman, R. (2008). Characterization of signal enhancement following the intraperitoneal injection of Gadolinium based contrast agents. Proc. Int. Soc. Mag. Reson. Med. 3206.

[60] Perles-Barbacaru, A.T., Berger, F., and Lahrech, H. (2013). Quantitative rapid steady state T1 magnetic resonance imaging for cerebral blood volume mapping in mice: Lengthened measurement time window with intraperitoneal Gd-DOTA injection. Magn. Reson. Med. 69, 1451–1456. 22760854 10.1002/mrm.24365

[61] Naffziger, H.C. (1924). Subdural fluid accumulations following head injury. J Am Med Assoc. 82, 1751–1752.

[62] Haines, D.E., Harkey, H.L., and Al-Mefty, O. (1993). The “subdural” space: a new look at an outdated concept. Neurosurgery 32, 111–120. 10.1227/00006123-199301000-00017 8421539

[63] Livingston, W.S., Gill, J.M., Cota, M.R., Olivera, A., O'Keefe, J.L., Martin, C., and Latour, L.L. (2017). Differential gene expression associated with meningeal injury in acute mild traumatic brain injury. J. Neurotrauma 34, 853–860. 27430610 10.1089/neu.2016.4479 PMC5314966

[64] Nabeshima, S., Reese, T., Landis, D.M., and Brightman, M. (1975). Junctions in the meninges and marginal glia. J. Comp. Neurol. 164, 127–169. 810497 10.1002/cne.901640202

[65] Mecheri, B., Paris, F., and Lübbert, H. (2018). Histological investigations on the dura mater vascular system of mice. Acta Histochem. 120, 846–857. 30292321 10.1016/j.acthis.2018.09.009

[66] Tu, T.-W., Williams, R.A., Lescher, J.D., Neekita, J., Turtzo, C., and Frank, J.A. (2016). Radiological-pathological correlation of diffusion tensor and magnetization transfer imaging in closed head traumatic brain injury model Ann. Neurol. 79, 907–920. 10.1002/ana.24641 PMC4887193 27230970

