# Supplementary material for: Meningeal and Visual Pathway Magnetic Resonance Imaging Analysis after Single and Repetitive Closed-Head Impact Model of Engineered Rotational Acceleration (CHIMERA)-Induced Disruption in Male and Female Mice
Source: J Neurotrauma. 2022 Jun 3;39(11-12):784–99. doi: 10.1089/neu.2021.0494 (PMC9225425; doi:10.1089/neu.2021.0494)

**Supplemental Figure 1**: Hand-drawn depiction of the ten ROIs (meninges=red, hippocampus=green, corpus callosum=dark blue, cerebellum=teal, brainstem=pink, optic tract=orange, lateral geniculate nucleus=dark green, superior colliculus=purple, muscle=yellow, cortex=gold) across several slices with estimated corresponding Allen Mouse Brain Atlas levels below for anatomical orientation.


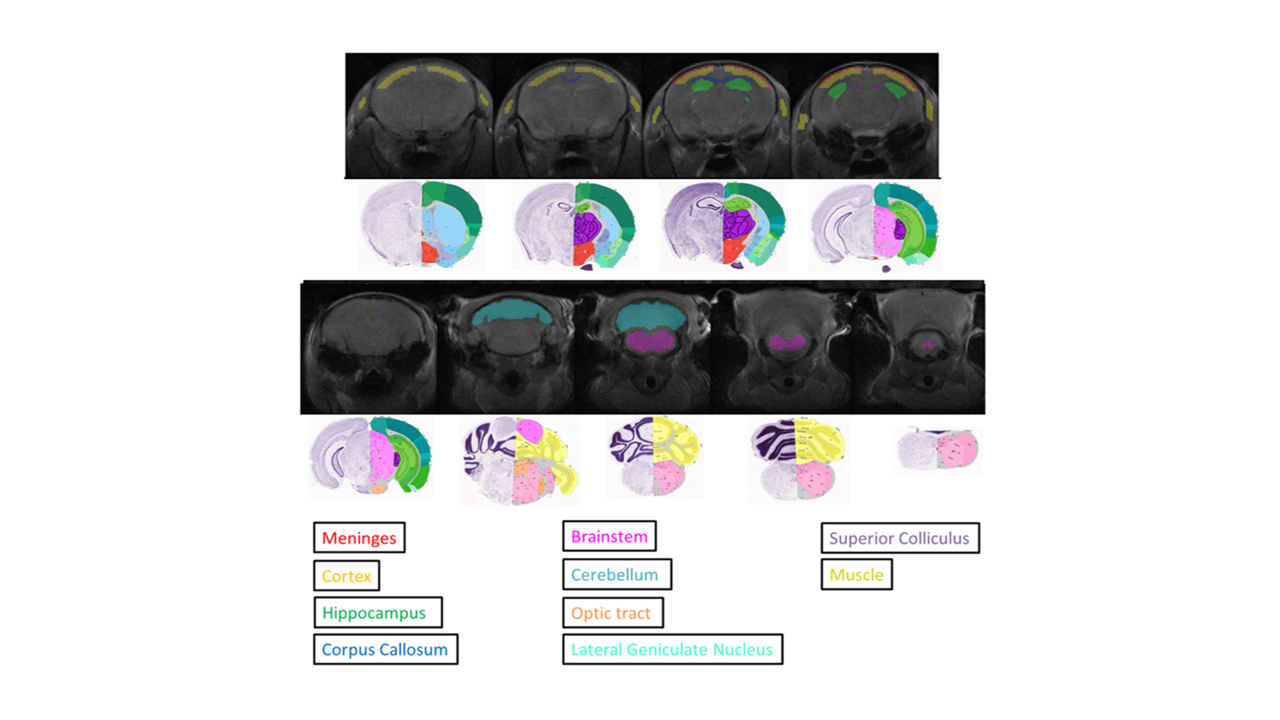

Supplement: Supplemental data [file Suppl_FigureS1.docx]
