# Supplementary material for: Meningeal and Visual Pathway Magnetic Resonance Imaging Analysis after Single and Repetitive Closed-Head Impact Model of Engineered Rotational Acceleration (CHIMERA)-Induced Disruption in Male and Female Mice
Source: J Neurotrauma. 2022 Jun 3;39(11-12):784–99. doi: 10.1089/neu.2021.0494 (PMC9225425; doi:10.1089/neu.2021.0494)
Supplement: Supplemental data [file Suppl_TableS3.docx]

**Supplemental Table 3**: Summary of all data for significant regions*

| Region | AUC | T2 | GFAP |
| --- | --- | --- | --- |
| Meninges | 1x male ↑ on Day 1 then ↓ on Day 7  4x ↑ on Day 1 and Day 7 | 1x ↑ on Day 1 | NA |
| Corpus Callosum | 1x CC male ↓ on Day 7 | ns | ns |
| Hippocampus | 1x male ↓ on Day 7 | ns | NA |
| Optic Tract | ns | 4x↑ on Day 1 then ↓ on Day 7 | 1x ↑ on Day 7  4x ↑ on Day 7 |
| Lateral Geniculate Nucleus | 1x male ↑ on Day 1 then ↓ on Day 7 | 4x ↓ on Day 7 | ns |
| Superior Colliculus | 1x male ↑ on Day 1 then ↓ on Day 7  1x female ↓ on Day 1 | ns | 4x ↑ on Day 7 |
| Brainstem | ns | 4x ↓ on Day 7 | NA |

*****Summary of all data for significant regions. Injury level severity, trend direction with respect to CHIMERA group, and day of interaction is provided for regions where significant changes were observed.
