# Supplementary material for: Meningeal and Visual Pathway Magnetic Resonance Imaging Analysis after Single and Repetitive Closed-Head Impact Model of Engineered Rotational Acceleration (CHIMERA)-Induced Disruption in Male and Female Mice
Source: J Neurotrauma. 2022 Jun 3;39(11-12):784–99. doi: 10.1089/neu.2021.0494 (PMC9225425; doi:10.1089/neu.2021.0494)

**Supplemental Figure 2**: Body weights across days for **A)** single and **B)** repetitive CHIMERA cohorts. Males had higher body weights compared to females, but there was no significant difference between injured and sham groups. The bars indicate means.


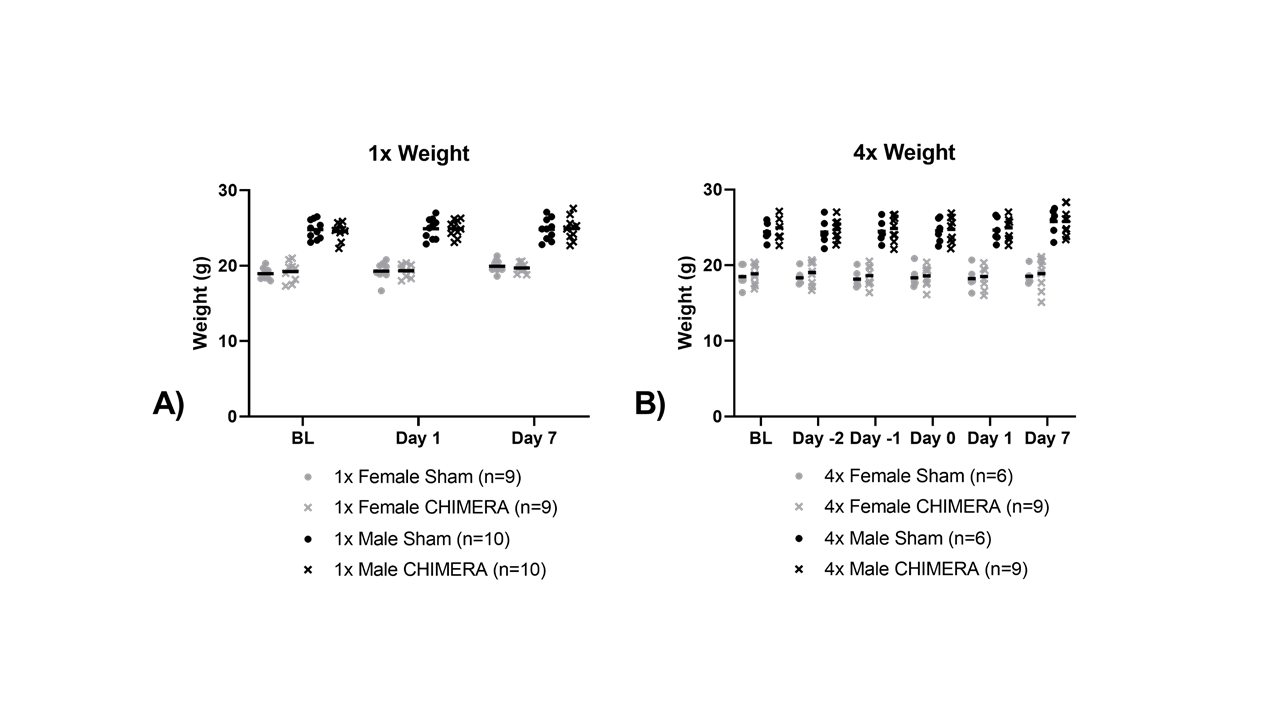

Supplement: Supplemental data [file Suppl_FigureS2.docx]
