# Supplementary material for: Meningeal and Visual Pathway Magnetic Resonance Imaging Analysis after Single and Repetitive Closed-Head Impact Model of Engineered Rotational Acceleration (CHIMERA)-Induced Disruption in Male and Female Mice
Source: J Neurotrauma. 2022 Jun 3;39(11-12):784–99. doi: 10.1089/neu.2021.0494 (PMC9225425; doi:10.1089/neu.2021.0494)
Supplement: Supplemental data [file Suppl_TableS1.docx]

**Supplemental Table 1**: Summary of AUC results for 1x CHIMERA and 4x CHIMERA, *p*-values from ANOVAs*

| AUC 4x CHIMERA Summary of *p*-values from ANOVAs | | | | | | | | | |
| --- | --- | --- | --- | --- | --- | --- | --- | --- | --- |
| Region | Injury | Sex | Day | Brain Region | Injury x Day (Day) | Brain Region x Sex | Injury x Sex x Day | Brain Region x Injury x Sex x Day |  |
| Meninges | ns | p < .001 | p = .013 | n/a | ns | n/a | p = .006  male 1x CHIMERA > 1x Sham on D1; p = .002  male 1x CHIMERA < 1x Sham on D1; p = .022 | n/a |  |
| Brain Regions | ns | p = .005 | p < .001 | p < .001 | p = .005 | p = .035 | ns | p = .018  Corpus Callosum: male 1x CHIMERA < 1x Sham on Day 7; p = .012  Hippocampus: male 1x CHIMERA < 1x Sham on Day 7; p = .008  Lateral Geniculate Nucleus: male 1x CHIMERA > 1x Sham on Day 1; p = .023  Male 1x CHIMERA < 1x Sham on Day 7; p = .002  Superior Colliculus: female 1x CHIMERA < 1x Sham on Day 1; p = .021  Male 1x CHIMERA > 1x Sham on Day 1; p = .024  Male 1x CHIMERA < 1x Sham on Day 7; p = .008 |  |
| Optic Tract | ns | ns | ns | n/a | ns | n/a | ns | n/a |  |
| AUC 4x CHIMERA Summary of *p*-values from ANOVAs | | | | | | | | | |
| Meninges | p = .014 | ns | p = .009 | n/a | p = .010  4x CHIMERA > 4x Sham on Day 1; p = .005  4x CHIMERA > 4x Sham on Day 7; p = .035 | n/a | ns | n/a |  |
| Brain Regions | ns | p = .041 | p < .001 | p < .001 | ns | p = .049  cerebellum | ns | ns |  |
| Optic Tract | ns | ns | ns | n/a | ns | n/a | ns | n/a |  |

*Abbreviations: n/a, not applicable; ns, not significant.
