# Supplementary material for: Meningeal and Visual Pathway Magnetic Resonance Imaging Analysis after Single and Repetitive Closed-Head Impact Model of Engineered Rotational Acceleration (CHIMERA)-Induced Disruption in Male and Female Mice
Source: J Neurotrauma. 2022 Jun 3;39(11-12):784–99. doi: 10.1089/neu.2021.0494 (PMC9225425; doi:10.1089/neu.2021.0494)
Supplement: Supplemental data [file Suppl_TableS2.docx]

**Supplemental Table 2**: Summary of T2 Relaxation Values for 1x CHIMERA and 4x CHIMERA, *p*-values from ANOVAs*

| T2 1x CHIMERA Summary of *p*-values from ANOVAs | | | | | | | | | |
| --- | --- | --- | --- | --- | --- | --- | --- | --- | --- |
| Region | Injury | Sex | Day | Brain Regions | Injury x Day (Day) | Sex x Day | Brain Region x Injury x Day | Injury x Sex x Day | Brain Region x Injury x Sex x Day |
| Meninges | ns | ns | ns | n/a | p = .009  1x CHIMERA > 1x Sham on Day 1; p = .010 | p = .032 | n/a | ns | n/a |
| Brain Regions | ns | ns | ns | p < .001 | ns | ns | Ns | ns | ns |
| T2 4x CHIMERA Summary of *p*-values from ANOVAs | | | | | | | | | |
| Meninges | ns | ns | ns | n/a | ns | ns | n/a | ns | n/a |
| Brain Regions | p = .037 | p = .001 | p = .019 | p < .001 | p < .001 | p = .001 | p < .001  Optic Tract: 4x CHIMERA > 4x Sham on Day 1; p < .001  4x CHIMERA < 4x Sham on Day 7; p < .001  Lateral Geniculate Nucleus: 4x CHIMERA < 4x Sham on Day 7; p = .036  Brainstem: 4x CHIMERA < 4x Sham on Day 7; p = .016 | p = .021 | ns |

*Abbreviations: n/a, not applicable; ns, not significant.
