# Supplementary material for: Meningeal and Visual Pathway Magnetic Resonance Imaging Analysis after Single and Repetitive Closed-Head Impact Model of Engineered Rotational Acceleration (CHIMERA)-Induced Disruption in Male and Female Mice
Source: J Neurotrauma. 2022 Jun 3;39(11-12):784–99. doi: 10.1089/neu.2021.0494 (PMC9225425; doi:10.1089/neu.2021.0494)
Supplement: Supplemental data [file Suppl_Data.docx]

**MRI data processing**

All registration steps were performed using the Advanced Normalization Tools software ^1^. All Bruker 2dseq files were converted to the NIFTI file format. An N4-bias field correction was applied to the first frame of the DCE data and the computed bias field was applied to the other DCE image frames ^2^. To correct for potential motion, frames 2 through 10 (Cohorts 1–2) or 20 (Cohorts 3–5) were rigidly registered to frame 1 using the Mattes mutual information algorithm as the cost function. The transformation was restricted to be within the acquisition plane (i.e., no through-plane motion was allowed). The first three image frames were averaged together and used to draw the ROIs.

To correct for small motions within the scan session, the T2-weighted post-contrast images were rigidly registered to the T2-weighted pre-contrast image using the mattes cost function. The transformation matrix was restricted to be within the image plane. The computed transformation matrices were applied to the post-contrast T2-relaxation map using cubic b-spline interpolation. An N4-bias field correction was applied to the 30 ms TE of the pre-contrast T2-weighted image set ^2^. The computed bias field was applied to all other TEs and TRs for the pre- and post-contrast T2-weighted images (all Cohorts). T2-relaxation maps were computed using a nonlinear fit in Matlab to solve for $S_{0}$ and $T_{2}$ in the following function:

$$S_{i}(\boldsymbol{x}_{j})=S_{0}(\boldsymbol{x}_{j})e^{-\frac{TE_{i}}{T_{2}(\boldsymbol{x}_{j})}}$$

where $S_{i}$ is signal intensity of the i^th^ TE of the j^th^ voxel, $S_{0}$ is the signal amplitude at a TE of 0 ms, and $T_{2}$ is the estimated relaxation parameter in ms.

Scans with low DCE values, three standard deviations below the mean baseline values, in the muscle ROI were identified as unsuccessful intraperitoneal gadolinium injections and were excluded. For unsuccessful Baseline or Day 7 injections, the scan was repeated on the following day. Ten animals from the 1x CHIMERA or 1x Sham groups received an additional scan or second gadolinium dose because of failed initial injections. In total 8/204 scans (1x Male Sham Day 1, 1x Male Sham Day 7, 1x Male CHIMERA Day 1, 1x Male CHIMERA Day 7, 4x Female CHIMERA Baseline, 4x Female CHIMERA Day 7, and two 4x Male Sham Day 7) were omitted due to death (2 scans), bad injections (5 scans), or ghost artifact (1 scan) discovered during image processing. To ensure outlier voxels did not bias the results, a Gaussian mixed model was applied to identify the number of voxel distributions present within each ROI of the Baseline scans; voxels outside ± 3 standard deviations from the mean of the estimated fit were excluded. The median value was then computed within each ROI for all metrics.

For DCE, the Area Under the Curve (AUC) was calculated to provide a more robust metric for image enhancement ^3^. The DCE percent enhancement (DCEPE) was calculated for each image frame using the formula for percent signal change from Baseline.

$$DCEPE_{i}\left( \boldsymbol{x}_{j} \right)=\frac{DCE_{i}\left( \boldsymbol{x}_{j} \right)-DCE_{pre}\left( \boldsymbol{x}_{j} \right)}{DCE_{pre}\left( \boldsymbol{x}_{j} \right)}*100$$

Where $DCE_{i}$ is the DCE signal in the j^th^ voxel of the i^th^ image frame and $DCE_{pre}$ (pre-contrast) is the signal in frame 2 of 10 (Cohorts 1–2) or 3 of 20 (Cohorts 3–5) of the j^th^ voxel. The AUC value was computed by applying the *cumtrapz* function in Matlab to the average DCEPE series in each ROI (frames 2–10 for Cohorts 1–2, and frames 3–20 for Cohorts 3–5). To account for variable amounts of contrast agent uptake across scans and animals, the AUC values in each ROI were normalized by the AUC value in the muscle ROI.

**Statistics (continued from Methods)**

T2 pre-contrast (T2 pre) is a clinically relevant metric for edema, impairment of the blood-brain barrier, and inflammation. However, when T2 post-contrast data was compared to T2 pre-contrast by T2 (i.e., (post-pre)/pre), changes in T2 post did not greatly differ from T2 pre values, so only T2 pre is reported.

The acquired scan data after 1x and 4x CHIMERA treatments were also analyzed for the brain with a four-factor linear mixed model (Brain Region x Injury x Sex x Day) with pertinent comparisons reported with Bonferroni correction. Preliminary inspection indicated the values for the mean AUC for the optic tract greatly differed compared to other brain regions. This structure was analyzed separately from the other brain ROIs.

Finally, as an estimate of the impact of CHIMERA on imaging values across the cohorts of animals that received one or repetitive injuries, Hedge’s *g* statistic^4^ was computed to estimate effect size using the following:

$g=\frac{{M_{1}-M}_{2}}{{SD}_{pooled}}$, where ${SD}_{pooled}=\sqrt{\frac{{{\left( n_{1}-1 \right)SD}_{1}^{2}+\left( n_{2}-1 \right)SD}_{2}^{2}}{n_{1}+n_{2}-2}}$, and $g\cong d\left[ 1-\frac{3}{4\left( n_{1}+n_{2} \right)-9} \right]$.

Where M refers to the expected means obtained from the analyses of variance, n_1_ is the sample size for sample_1_ and s_1_ is the standard deviation for mean_1_. n_2_ and s_2_ are the sample size and standard deviation for sample_2_, respectively. *g* is the unbiased version of Cohen’s *d* ^5^.

**Additional brain region analyses**

Evaluation of brain regions after 1x CHIMERA with a four-factor (Brain Region x Injury x Sex x Day) interaction (*p* = .018) suggested the corpus callosum (*p* = .012), hippocampus (*p* = .008), lateral geniculate nucleus (LGN, Day 1 *p* = .023, Day 7 *p* = .002), and superior colliculus (Day 1 *p* = .024, Day 7 *p* = .008) exhibited DCE changes in male mice. Only the superior colliculus displayed a significant decrease in AUC in injured females on Day 7 (*p* = .021). Three-factor interactions for T2 brain analyses appeared with Brain Region x Injury x Day (*p* < .001) and Injury x Sex x Day (*p* = .021). However, several regions had lower T2 pre values one week after 4x CHIMERA including the optic tract (OPT, Day 1 *p* < .001, Day 7 *p* < .001), LGN (*p* = .036), and brainstem (*p* = .016). GFAP quantification showed astrogliosis changes only in the OPT for 1x CHIMERA (*p* = .0100) and 4x CHIMERA (*p* = .0284) and superior colliculus (*p* = .0001) after 4x CHIMERA.

**Supplement Cited References**

1. Avants, B.B., Tustison, N.J., Stauffer, M., Song, G., Wu, B. and Gee, J.C. (2014). The Insight ToolKit image registration framework. Front Neuroinform. 8, 44.

2. Tustison, N.J., Avants, B.B., Cook, P.A., Zheng, Y., Egan, A., Yushkevich, P.A. and Gee, J.C. (2010). N4ITK: improved N3 bias correction. IEEE Trans Med Imaging 29, 1310-1320.

3. Iliff, J.J., Lee, H., Yu, M., Feng, T., Logan, J., Nedergaard, M. and Benveniste, H. (2013). Brain-wide pathway for waste clearance captured by contrast-enhanced MRI. J Clin Invest. 123, 1299-1309.

4. Hedges, L.V. (1981). Distribution theory for Glass's estimator of effect size and related estimators. J Educ Stat. 6, 107-128.

5. Ellis, P.D. (2009). Effect size calculators. [**https://www.polyu.edu.hk/mm/effectsizefaqs/calculator/calculator.html**](https://www.polyu.edu.hk/mm/effectsizefaqs/calculator/calculator.html)
